# Loss of sfrp1 promotes ductal branching in the murine mammary gland

**DOI:** 10.1186/1471-213X-12-25

**Published:** 2012-08-28

**Authors:** Kelly J Gauger, Akihiko Shimono, Giovanna M Crisi, Sallie Smith Schneider

**Affiliations:** 1 Pioneer Valley Life Sciences Institute, Baystate Medical Center, Springfield, MA, 01199, USA; 2 Biology Department, University of Massachusetts, Amherst, MA, 01003, USA; 3 TransGenic Inc, Chuo, Kobe, 650-0047, Japan; 4 Department of Pathology, Baystate Medical Center, Springfield, MA, 01199, USA; 5 Veterinary and Animal Sciences, University of Massachusetts, Amherst, MA, 01003, USA; 6 Pioneer Valley Life Sciences Institute, 3601 Main St, Springfield, MA, 01199, USA

**Keywords:** SFRP1, Mammary gland, Branching morphogenesis

## Abstract

**Background:**

Secreted frizzled-related proteins (SFRPs) are a family of proteins that block the Wnt signaling pathway and loss of SFRP1 expression is found in breast cancer along with a multitude of other human cancers. Activated Wnt signaling leads to inappropriate mammary gland development and mammary tumorigenesis in mice. When SFRP1 is knocked down in immortalized non-malignant mammary epithelial cells, the cells exhibit a malignant phenotype which resembles the characteristics observed in metastatic breast cancer stem-like cells. However, the effects of SFRP1 loss on mammary gland development in vivo are yet to be elucidated. The work described here was initiated to investigate the role of SFRP1 in mammary gland development and whether SFRP1^−/−^ mice exhibit changes in mammary gland morphology and cell signaling pathways shown to be associated with SFRP1 loss in vitro.

**Results:**

10 week old nulliparous SFRP1^−/−^ mammary glands exhibited branching with clear lobulo-alveolar development, which normally only occurs in hormonally stimulated mid-pregnant wt mammary glands. Explant cultures of SFRP1^−/−^ mammary glands display increased levels of a well known Wnt signaling target gene, Axin2. Histomorphologic evaluation of virgin glands revealed that by 10 weeks of age, the duct profile is markedly altered in SFRP1^−/−^ mice showing a significantly higher density of ducts with distinct alveoli present throughout the mammary gland, and with focal ductal epithelial hyperplasia. These findings persist as the mice age and are evident at 23 weeks of age. Changes in gene expression, including c-Myc, TGFβ-2, Wnt4, RANKL, and Rspo2 early in mammary gland development are consistent with the excessive hyper branching phenotype. Finally, we found that loss of SFRP1 significantly increases the number of mammary epithelial cells capable of mammosphere formation.

**Conclusions:**

Our study indicates that SFRP1 gene is critical for maintaining proper mammary gland development, and that reduced levels of SFRP1 results in hyperplastic lesions and its loss may be a critical event in cancer initiation.

## Background

Members of the Wnt family of secreted proteins play crucial roles in the morphogenesis of most organ systems and regulate cellular proliferation, differentiation, migration and apoptosis [1-3]. The best characterized Wnt pathway is the canonical Wnt/β-catenin pathway whereby β-catenin stimulates the expression of specific target genes including the c-Myc oncogene. Atypical activation of the Wnt/β-catenin signaling pathway contributes to the genesis of a wide range of human cancers, including breast cancer [3]. In the murine breast, aberrantly activated Wnt signaling leads to inappropriate mammary gland development and mammary tumorigenesis [1].

In addition to embryonic development, Wnt family members are common regulators of postnatal development and of homeostasis in adult tissues, including the growth and differentiation of the mammary gland [4-6]. The first stage of mammary gland development in mice is the specification of mammary rudiments, a prenatal event which occurs around embryonic day 10. The second stage of mammary development begins at around 3 weeks of age at the onset of puberty in response to ovarian hormones, resulting in a rapid expansion of the preexisting rudimentary ductal tree [7]. Branching ductal morphogenesis proceeds across the entire mammary fat pad and is completed at approximately 10 weeks of age. During this phase of development, the growing tips of the ducts form the highly proliferative terminal end buds (TEBs), which house mammary stem cells [8,9]. The major changes in morphology and function that are initiated at the start of pregnancy constitute a third stage in mammary gland development that is influenced and regulated by Wnt signaling. Development of the gland at this stage can be reduced to two principal elements: formation of extensive secondary branches of the ductal tree, and the appearance of alveolar units that constitute the secretory apparatus of the gland. Pregnancy dependent hormones normally trigger both of these aspects of mammary morphogenesis, but both can also be induced prematurely by ectopic expression of Wnt proteins in virgin mice [9-12].

Secreted frizzled-related proteins (SFRPs) are a family of Wnt antagonists which contain a cysteine-rich domain that is homologous to the Wnt-binding domain of frizzled receptor proteins [13]. However, SFRPs do not contain a transmembrane domain and therefore are released into the extracellular compartment where they antagonize Wnt signaling by binding to Wnt ligands and preventing ligand-receptor interactions and signal transduction [14]. Loss of SFRP1 expression is found in multiple human cancers including breast cancer [15-17]. When SFRP1 is knocked down in immortalized non-malignant mammary epithelial cells, the cells (TERT-siSFRP1) acquire a malignant phenotype characteristically observed in metastatic breast cancer stem-like cells [18,19]. The work described here was initiated to investigate the role of SFRP1 in mammary gland development and whether SFRP1^−/−^ mice exhibit changes in mammary gland morphology and cell signaling pathways shown to be associated with SFRP1 loss in vitro. Previously, analysis of 41 different non-skeletal tissues, including the mammary gland, in 20 or 40 week old mice has shown no overt morphologic differences between SFRP1^+/+^ and SFRP1^−/−^ animals [20]. However, considering that mammary gland development occurs around puberty, we chose to look at the morphological effects of SFRP1 loss beginning at this critical development stage, and at an adult stage. The significance of these studies is considerable because methylation of SFRP1 (resulting in suppression of SFRP1 expression) has been demonstrated to be an early change in premalignant breast lesions [21,22].

## Results and discussion

### SFRP1^−/−^ mice exhibit precocious mammary gland development

**Figure 1:**
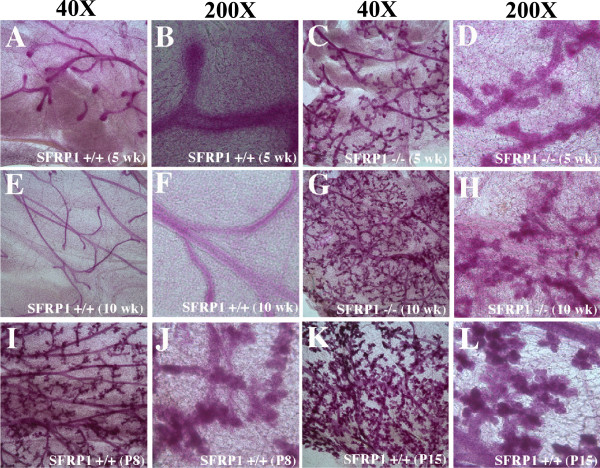
**Comparison of the morphology of 5 wk virgin, 10 wk virgin, pregnant day 8, and pregnant day 15 mammary glands.** Whole mounts of inguinal mammary glands stained with carmine alum from (**A**-**B**) 5 wk SFRP1^+/+^, (**C**-**D**) 5wk SFRP1^−/−^, (**E**-**F**) 10 wk SFRP1^+/+^, (**G**-**H**) 10 wk SFRP1^−/−^, (**I**-**J**) wt P8, and (**K**-**L**) wt P15 females. (**B**,**D**,**F**,**H**,**J**,**L**) Shown medium magnification (200X) of selection from adjacent low magnification left panel (40X). Tissue was harvested from and assessed in 6 animals/genotype.

**Figure 2:**
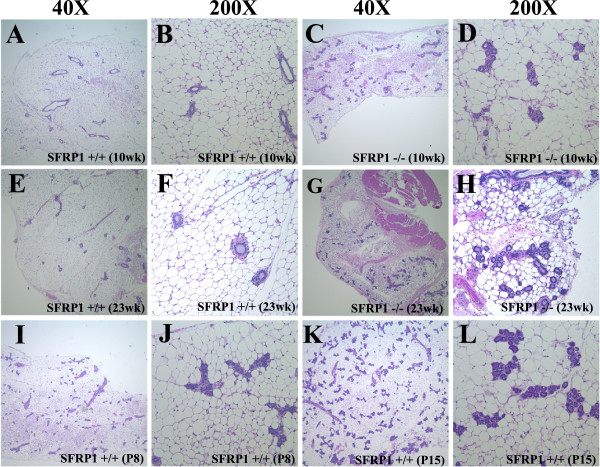
**Hematoxylin/eosin-stained sections of 10 wk virgin, 23 wk virgin, pregnant day 8, and pregnant day 15 mammary glands.** 5 μm section from (**A**,**B**) 10 wk SFRP1^+/+^ female, (**C**,**D**) 10 wk SFRP1^−/−^female, (**E**,**F**) 23 wk SFRP1^+/+^ female, (**G**,**H**) 23 wk SFRP1^−/−^female, (**I**,**J**) wt P8 female, and (**K**,**L**) wt P15 female. (A,C,E,G,I,K) Low magnification (40X) images illustrate the significant increase in the number of ducts and alveoli present in 10 wk virgin SFRP1^−/−^, 23 wk virgin SFRP1^−/−^, P8 SFRP1^+/+^,and P15 SFRP1^+/+^ when compared with virgin SFRP1^+/+^ animals. Tissue was harvested from and assessed in 6 animals/genotype.

To determine the effect of SFRP1 loss on early mammary gland development, we examined whole mount preparations of mammary glands from female virgin SFRP1^+/+^ and SFRP1^−/−^mice and compared our findings with pregnant day 8 (P8) and P15 wt animals (Figure 1). Differences in the mammary gland morphology could be detected between SFRP1^+/+^ and SFRP1^−/−^ mice at early stages of puberty. At 5 weeks of age, SFRP1^+/+^ mammary glands exhibited a branching ductal structure typical of a virgin female with very few alveoli growing from terminal branches at the end buds of the ducts (Figure 1A). At higher magnification, the branching ductal structures displayed a smooth surface with a 1–2 cell lining (Figure 1B). In contrast, in 5 weeks old SFRP1^−/−^ animals the mammary gland displayed evidence of nascent ductal side branching (Figure 1C-D), and at 10 weeks of age a significantly more complex arborized ductal network was formed as compared to the age-matched SFRP1^+/+^ gland (Figure 1E-H). In addition, SFRP1^−/−^ mammary glands exhibited branching with clear lobulo-alveolar development (low and high magnification images, Figure 1G-H), which normally only occurs in hormonally stimulated mid-pregnant wt mammary glands (Figure 1I-L). Histomorphologic evaluation of virgin glands revealed that at 5 weeks of age, there is only a marginal difference in the number of ducts present in SFRP1^−/−^ animals as compared to SFRP1^+/+^ animals (data not shown). By 10 weeks of age the duct profile is markedly altered in SFRP1^−/−^ mice showing a significantly higher density of ducts with distinct alveoli present throughout the mammary gland (Figure 2), and with focal ductal epithelial hyperplasia. This pattern of ductal hyperplasia and precocious alveolar formation that occurs in the 10 week virgin SFRP1^−/−^ mammary gland (Figure 2C-D) resembles that of the wt P8 ductal profile (Figure 2I-J). Moreover, these observations persist as the SFRP1^−/−^ mice age. While alveoli are suitably still absent in 23 week virgin SFRP1^+/+^ animals (Figure 2E-F), the presence of precocious alveoli remains and even expands in 23 week virgin SFRP1^−/−^ mice (Figure 2G-H), with mammary gland proliferation baring resemblance to wt P15 mice (Figure 2K-L). While mammary glands have previously been examined in SFRP1^+/+^ and SFRP1^−/−^ 20 and 40 week old animals [20], the methodology of mammary gland analysis we employed revealed that there is a striking difference in morphology between geneotypes. Our findings of precocious development and persistent hyperplasia of ducts/lobulo-alveolar units in SFRP1^−/−^ mice are significant because they suggest that loss of SFRP1 may contribute to an increase in breast tumor susceptibility.

**Figure 3:**
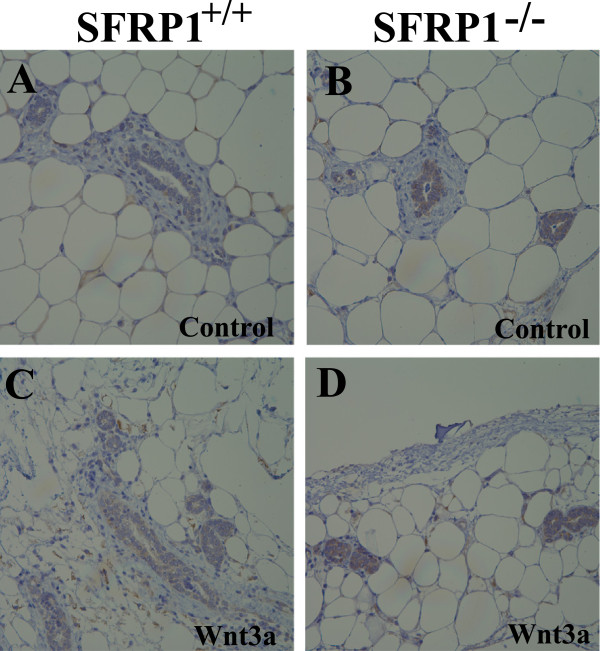
**Protein expression analysis of the Axin2 ex vivo*.*** Mouse mammary gland sections were subjected to immunohistochemistry and stained for the Wnt target gene, Axin2 (brown chromogen). Representative ducts are shown from explant mammary gland tissues derived from SFRP1^+/+^ and SFRP1^−/−^ mice cultured in control media or media containing an exogenous Wnt ligand (Wnt3a) to induce Wnt signaling: (**A**) SFRP1^+/+^ in control media, (**B**) SFRP1^−/−^ in control media, (**C**) SFRP1^+/+^ in Wnt3a media, (**D**) SFRP1^−/−^ in Wnt3a media. Tissue was harvested from and assessed in 6 animals/genotype.

Our results are consistent with previous studies showing that upregulation of the Wnt/β-catenin pathway and activation of β-catenin in mice induces precocious lobulo-alveolar hyperplasia [10-12]. In particular, Lane *et. al.* demonstrated that mammary glands from Wnt-10b virgin mice are more branched and have a higher density of ducts compared to non-transgenic littermates [11]. This pattern of ductal hyperplasia and precocious alveolar formation has also been described in MMTV-Wnt1 mice [9,23]. Moreover, Bradbury *et. al.* showed that constitutive expression of Wnt4 in the virgin mammary gland also induces structures with a morphology similar to that seen in pregnancy [24]. To determine whether the mammary glands of SFRP1^−/−^ mice have more responsive Wnt/β-catenin signaling, we exploited an ex vivo tissue culture assay to survey the expression of a well known Wnt/β-catenin target gene, Axin2, in response to Wnt3a. As shown in Figure 3, both in the absence and presence of the Wnt3a β-catenin stimulating ligand, SFRP1^−/−^ mice express higher levels of Axin2 protein (Figure 3 B&D). Taken together, our results suggest that SFRP1 is an essential regulator of normal mammary gland development and likely functions in part by antagonizing the Wnt/β-catenin signaling pathway.

### Genes involved in mammary gland morphogenesis are overexpressed early in SFRP1^−/−^ mice

**Figure 4:**
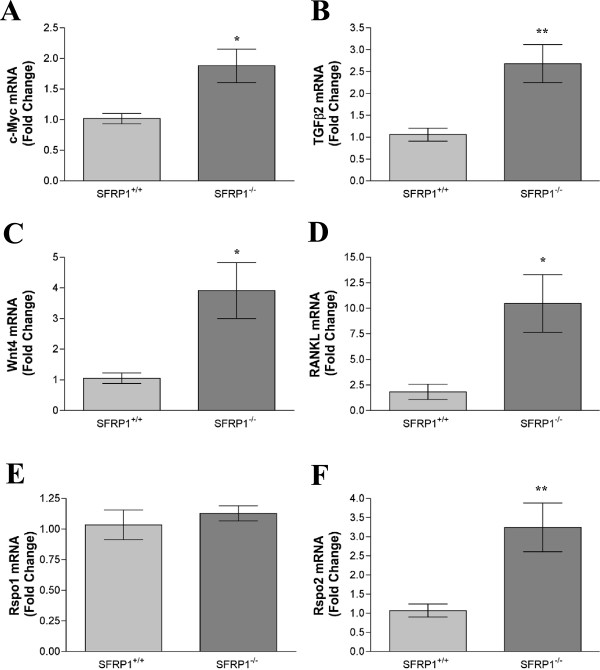
**Expression analysis of genes altered in SFRP1**^**−/−**^**animals that may be involved in mammary gland branching.** For real-time PCR analysis of c-Myc, TGF-β2, Wnt4, RANKL, Rspo1, and Rspo2 gene expression, total RNA was isolated from the mammary glands of 5wk virgin SFRP1^+/+^ and SFRP1^−/−^ females (n = 6 animals/genotype). Real-time PCR experiments were carried out in duplicate. The results shown represent experiments performed in duplicate and normalized to the amplification of β-actin mRNA. Bars represent mean ± SEM of the fold change with respect to vehicle treated cells. *p < 0.05, **p < 0.01 (significantly different from vehicle treated cells using student’s *t*-test).

We next sought to determine whether there were changes in gene expression early in development that might help explain the excessive hyper branching phenotype observed in 10 week old SFRP1^−/−^ mice. c-Myc is a well characterized downstream gene of Wnt/β-catenin signaling that is upregulated in response to β-catenin transcriptional activation. Real-time PCR studies confirmed that c-Myc mRNA levels were significantly higher in the mammary gland of SFRP1^−/−^mice (Figure 4A). Interestingly c-Myc has been shown to induce precocious mammary development and transformation when overexpressed in the mouse mammary gland [25,26]. In addition, virgin mice overexpressing constitutively active β-catenin express abnormally high levels of c-Myc mRNA [10].

Transforming growth factor (TGF)-β is a multifunctional cytokine that regulates a variety of physiological processes and also plays a dual role during mammary gland development and tumorigenesis. We have shown that loss of SFRP1 expression in vitro allows mammary cells to acquire sensitivity to TGF-β signaling [27]. In an initial screen of genes affected by SFRP1 loss in HMECs we identified several upregulated genes within the TGF-β pathway, including the TGF-β2 ligand [27]. The real-time PCR data presented here demonstrate that SFRP1 loss in vivo also results in significantly increased mRNA levels of TGF-β2 (Figure 4B). These findings may in part explain the altered mammary gland morphogenesis observed in SFRP1^−/−^ mice. Specifically, it has been shown that TGF-β2, but not TGF-β1 or TGF-β3, is critical for lung branching in vivo [28] and synergizes with Wnt to promote mammary gland branching in vitro [29].

As discussed previously, overexpression of Wnt1, Wnt10b, and/or Wnt4 induces mammary gland hyper branching. Therefore, we wanted to establish whether these particular Wnt ligands are upregulated in SFRP1^−/−^ animals. The expression of Wnt1 and Wnt10b was unaffected by SFRP1 loss (data not shown), however Wnt4 mRNA levels were significantly elevated in the mammary gland of SFRP1^−/−^ mice (Figure 4C). Interestingly, Wnt4 is expressed during the period when side-branching occurs in early to mid-pregnancy [30,31]. Brisken *et. al.* showed that Wnt4 null mammary glands were deficient in early lobulo-alveolar mammary outgrowth during pregnancy, and that Wnt4 is an effector for progesterone-induced mammary growth [32].

Critical to the Wnt4 downstream signaling for branching morphogenesis is the receptor of activated NF-κB ligand (RANKL) [33,34]. RANKL was originally characterized for its role in the development, survival, and activation of osteoclasts during bone remodeling [33]. Subsequent studies have shown that RANKL deficient mice exhibit a significant decrease in parity-induced mammary alveologenesis which results in a lactational defect [34]. Furthermore, transgenic overexpression of RANKL or RANK (RANKL receptor) alone into the murine mammary gland elicits ductal side branching, alveologenesis, and mammary hyperplasia [35,36]. Considering that SFRP1 has been shown to bind to and inhibit RANKL mediated action [37], we sought to determine whether the expression of RANKL is affected by SFRP1 loss. Indeed, we found that mRNA levels of RANKL were significantly elevated in the mammary gland of SFRP1^−/−^ mice (Figure 4D). These data lend support to the notion that SFRP1 may play a role in tumor susceptibility since abrogation or accentuation of RANKL signaling renders the mammary epithelium markedly resistant or susceptible to mammary tumorigenesis respectively [36,38]. Since RANKL has a role in bone remodeling and loss of SFRP1 increases the thickness of the trabecular bones [20], it has been suggested that SFRP1 inhibitors may be beneficial for the treatment and/or prevention of osteoporosis. However, SFRP1 inhibitors may not be beneficial to women who are genetically predisposed or who have pre-malignant lesions and may put them at risk for developing breast cancer.

R-spondin (RSPO) family proteins activate the canonical WNT signaling pathway by binding to the Wnt co-receptor LRP6 leading to β-catenin-dependent gene activation [39-41]. Interestingly, the expression of Rspo2 and other Rspo family members is induced when mouse mammary tumor virus (MMTV) is inserted into mammary epithelial cells, and these proteins have been associated with malignant transformation and tumorigenesis [42-44]. Loss of Rspo1 hinders the development of the murine mammary gland leading to lack of side branching along secondary ducts [45]. At 5 weeks of age in SFRP1^-/-^ mice, we started observing changes in the expression of genes involved in branching morphogenesis. Although we did not see Rspo1 changes at this stage (Figure 4E), the expression of Rspo2 is significantly elevated (Figure 4F). Additionally enhanced side branching at 10 wks of age in response to SFRP1 loss is associated with significantly elevated expression of both Rspo1 and Rspo2 (data not shown). These findings may partially explain how SFRP1 loss increases the expression of Wnt4, through Rspo2 enhancement of the β-catenin-dependent activity of Wnt4 in murine epithelial cells [46]. Furthermore, the expression pattern of Rspo1 parallels that of Wnt4 during mammary gland development which indicates that there is functional relationship between these Wnt signaling proteins [45].

### Mammary epithelial cells derived from SFRP1^−/−^ mice have more mammosphere initiating cells

Ductal outgrowth is driven by TEBs, which are believed to house a small population of cells that are essential for mammary gland development, mammary stem cells (MaSCs) [8,9]. Importantly, MaSCs are capable of regenerating all cell-lineages that comprise the murine mammary gland and support for their existence comes from cell enrichment studies using specific cell surface markers as well as mammary gland transplantation experiments [47,48]. Similar to stem cells in either the hematopoietic system or neuronal system, the self-renewal and differentiation of MaSCs is regulated by multiple genes and pathways. Several studies have shown that Wnt/β-catenin signaling is activated in several stem cell types, including in mammary stem cells. Liu *et. al.* demonstrated that transgenic mice with activated Wnt/β-catenin signaling in the mammary gland have a significantly higher stem cell-enriched population compared to wild type mice [49]. Additionally, the stem cell-enriched population of cultured primary mammary epithelial cells was significantly increased when cells were treated with a Wnt agonist and this effect could be abrogated by the addition of a Wnt pathway inhibitor [49].

**Figure 5:**
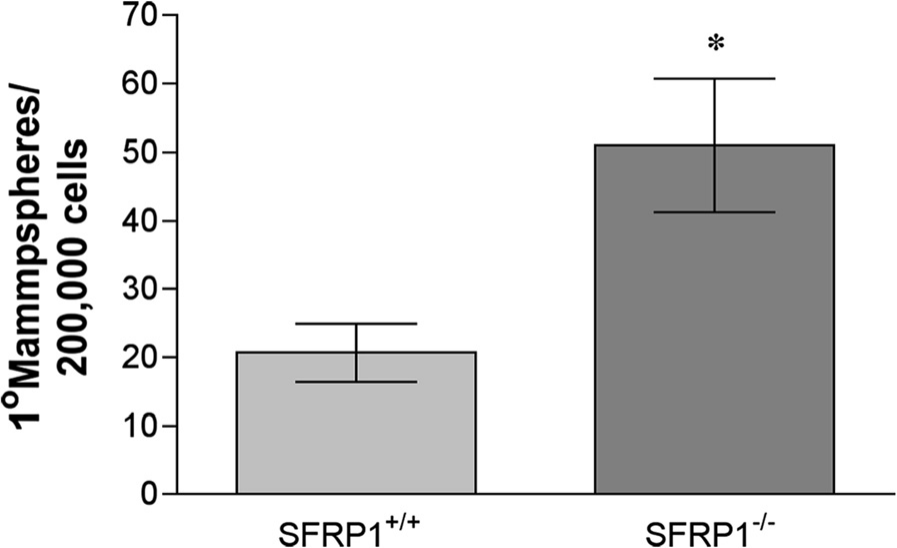
**Loss of SFRP1 promotes the expansion of mammosphere initiating cells.** Mouse mammary epithelial cells from SFRP1^+/+^ and SFRP1^-/-^ (n = 4 animals/genotype) were seeded on low attachment plates and allowed to grow for 21 days. In vitro quantification of mammpospheres formed by cells as described. Bars represent mean ± SEM number of spheres formed/20,000 seeded cells from 3 separate experiments. *p < 0.05 (significantly different from SFRP1^+/+^ MMEC cells using student’s *t*-test).

Stem cells and progenitor cells from the human mammary gland are able to grow in an anchorage independent manner (constituting mammospheres) and have been shown to express very low levels of SFRP1 [50]. Interestingly, the expression of SFRP1 is up-regulated when mammospheres are induced to differentiate [50]. Considering the premature branching that occurs in the SFRP1^−/−^murine mammary gland and that SFRP1 is a Wnt signaling antagonist, mammosphere formation capacity was compared between mammary epithelial cells derived from SFRP1^+/+^ and SFRP1^−/−^ animals. We found that loss of SFRP1 significantly increases the number of mammary epithelial cells capable of mammosphere formation (Figure 5). These data are fully consistent with previous studies showing that inhibition of SFRP1 increases mammosphere formation [18,19]. Although mammosphere formation is not a definitive measure of the stem cell population, the literature has shown that such a capacity is constantly associated with resolute in vivo stem cell measures [51-53]. If SFRP1 loss indeed enhances stem cell number, the increase in mammosphere formation is of importance because stem cells are thought to play a key role in malignant transformation of the breast since they are more likely to accumulate mutations and pass them to progeny during their long-life span [54,55]. Therefore the potential effect of SFRP1 loss on stem cell number and mammosphere formation is critical to breast tumorigenesis.

## Conclusions

The work described herein clearly demonstrate that SFRP1^−/−^ mice exhibit precocious mammary gland side branching with clear lobulo-alveolar development, which normally only occurs in hormonally stimulated mid-pregnant wt mammary glands. These changes may be partially explained by alterations in the expression of genes critical for mammary gland development and by an increase in the number of mammosphere forming cells. Previously, it has been demonstrated that suppression of SFRP1 expression is an early change in human premalignant breast lesions [21]. Taken together, our study indicates that the SFRP1 gene is critical for maintaining proper mammary gland development, that reduced levels of SFRP1 results in hyperplastic lesions, and its loss may be a critical event in cancer initiation. Therefore, caution should be exercised in the potential use of SFRP1 inhibitors for the treatment of osteroporosis until the effects of such inhibition on breast tumorigenesis are fully elucidated.

## Methods

### Animals

All procedures were performed in accordance with the NIH guidelines for the ethical treatment of animals and were approved by the Baystate Medical Center Institutional AnimalCare and Use Committee before initiating these studies. Female 129/C57Blk6 mice (n = 6) were individually housed in plastic cages with food and water provided continuously, and maintained on a 12:12 light cycle.

### Whole-mounts and carmine stain of mammary glands

Mice were euthanized with carbon dioxide and fifth inguinal mammary glands were excised from 5 wk virgin, 10 wk virgin, pregnant day 8, and pregnant day 15 animals, spread on microscope slides, and fixed overnight in methacarn (60% methanol, 30% chloroform, 10% acetic acid). The fixed glands were washed in 70% ethanol for 15 min, rinsed in water for 5 min, and stained overnight at 4°C in carmine alum stain (1 g carmine and 2.5 g aluminum potassium sulfate in 500 ml water). The glands were then dehydrated progressively in 70%-95%-100% ethanol, cleared in xylene for 1 hr, and mounted on slides with Cytoseal™XYL mounting medium (Richard-Allan Scientific). Mammary whole mounts where photographed using an Olympus BX41 light microscope using SPOTSOFTWARE (Diagnostic Instruments, Inc, Sterling Heights, MI).

### Histomorphology

Fourth or sixth inguinal mammary glands from 5 wk virgin, 10 wk virgin, 23 wk virgin, pregnant day 8, and pregnant day 15 animals (n = 6 animals/genotype) were formalin-fixed and paraffin-embedded. Tissue sections were cut on a Leica microtome at a thickness of 4 μm on Superfrost-plus slides, and stained with hematoxylin and eosin (H&E) per standard protocol. Briefly, slides were dried in a microwave for 1 min and then at 62°C for 15 minutes. Slides were subsequently de-paraffinized with xylene 3X, cleared with graded ETOH (100% *X*2, 95%, 70%), and rinsed in dH_2_O. Sections were stained for 3 minutes in Mayer’s hematoxylin, washed with glacial acetic acid water for 15 sec, and then ammonia water to blue, and stained with eosin. Finally, slides were dehydrated in ETOH and xylene before manual coverslipping. Stained images were captured with an Olympus BX41 light microscope using SPOTSOFTWARE.

### Ex vivo tissue culture and immunohistochemistry

Mice were euthanized with carbon dioxide and fourth inguinal mammary glands were excised (n = 6 animals/genotype). The tissue was aseptically minced and for each genotype, a representation from all 6 animals was placed on 2 different Surgifoam gelatin sponges (Ferrosan, Sueborg, Denmark) in 60 mm tissue culture dishes containing 5 mL of control or Wnt3a medium prepared as described [18]. Explant cultures were maintained for 24 hours in 5% CO_2_ air and subsequently formalin-fixed and paraffin-embedded.

Immunohistochemistry (IHC) was performed on a DakoCytomation autostainer using the Envision HRP Detection system (Dako, Carpinteria, CA). Each mammary tissue block was sectioned at 4 μm on a graded slide, deparaffinized in xylene, rehydrated in graded ethanols, and rinsed in Tris-phosphate-buffered saline (TBS). Heat induced antigen retrieval was performed in a microwave at 98°C in 0.01 M citrate buffer. After cooling for 20 minutes, sections were rinsed in TBS and subjected to the primary rabbit polyclonal anti-Axin2 antibody (1:100, Abcam, ab32197) for 45 minutes. Immunoreactivity was visualized by incubation with chromogen diaminobenzidine (DAB) for 5 minutes. Tissue sections were counterstained with hematoxylin, dehydrated through graded ethanols and xylene, and cover-slipped. Images were captured with an Olympus BX41 light microscope using SPOTSOFTWARE.

### RNA isolation and real-time PCR analysis

Total RNA was extracted from the sixth inguinal mammary glands of 5 week old animals using an acid-phenol extraction procedure [56], according to the manufacturer’s instructions (Trizol, Invitrogen, Carlsbad, CA). Relative levels of the mRNA expression of target genes was determined by quantitative real-time PCR using the M3005P™ real-time PCR system (Stratagene, La Jolla, CA) and all values were normalized to the amplification of β-Actin. The PCR primer sequences are described in Table 1. The assays were performed using the 1-Step Brilliant^®^ SYBRII^®^ Green QRT-PCR Master Mix Kit (Stratagene) containing 200 nM forward primer, 200 nM reverse primer, and 100 ng total RNA. The conditions for cDNA synthesis and target mRNA amplification were performed as follows: 1 cycle of 50°C for 30 min;1 cycle of 95°C for 10 min; and 35 cycles each of 95°C for 30 s, 55°C for 1 min, and 72°C for 30 s.

**Table 1:** PCR primer sequences

		
cMyc	forward	5’-CAGCGACTCTGAAGAAGAGC-3’
	reverse	5’-GTTGTGCTGGTGAGTGGAGA-3’
Wnt-4	forward	5’-ATGCCCTTGTCACTGCAAA-3’
	reverse	5’-CTGGACTCCCTCCCTGTCTT-3’
TGF-β2	forward	5’-AAGCTTCGGGATTTATGGTG-3’
	reverse	5’-TGGAGTTCAGACACTCAAA-3’
RANKL	forward	5’-TGAAGACACACTACCTGACTCCTG-3’
	reverse	5’-CCACAATGTGTTGCAGTTCC-3’
Rspo1	forward	5’- CGACATGAACAAATGCATCA -3’
	reverse	5’- CTCCTGACACTTGGTGCAGA -3’
Rspo2	forward	5’- GCCCATCAGGGTATTATGGA -3’
	reverse	5’- TCACAGTTTTCTATTCTGCATCG -3’
β-Actin	forward	5’- CTAAGGCCAACCGTGAAAAG -3’
	reverse	5’- ACCAGAGGCATACAGGGACA -3’

### Primary mouse mammary cell isolation and mammosphere culture

Eight 8–10 week old virgin mice were euthanized with carbon dioxide (4 SFRP1^+/+^ and 4 SFRP1^−/−^) and fourth mammary glands were harvested, minced, and finally dissociated in DMEM:F12 (Sigma, St. Louis, MO) supplemented with 5 % fetal bovine serum (Gibco, Paisleley, UK), 2 mg/ml collagenase (Worthington Biochemicals, Freehold, NJ), 100u/ml hyaluronidase (Sigma), 100u/ml pen/strep (Gibco) and 100 μg/ml gentamicin (Gibco) for 6 hours. The cell pellet was collected and further dissociated with 1mlpre-warmed 0.05% Trypsin-EDTA (Gibco) and 200μl1mg/ml Dnase I (Roche, Mannheim, Germany). Cell suspensions were sieved through a 40 μm cell strainer to obtain single cell suspensions. Primary single cells were seeded onto ultra-low attachment dishes (Corning, Corning, NY) at a density of 20,000 viable cells/ml. Cells were grown in a serum-free mammary growth medium (EpiCult®B for Mouse Mammary Epithelial Cell Culture, Vancouver, BC) supplemented with 10ng/ml EGF (Sigma), 10 ng/ml FGF (Sigma), 4 μg/ml heparin, 100u/ml pen/strep (Gibco) and 100 μg/ml gentamicin (Gibco) [50]. On day 21, the total number of mammospheres was quantified by counting spheres that were at least 100 μm in size. Images of mammosphere formation were captured with a Nikon Eclipse TE2000-Uusing Metaview™ software.

## Abbreviations

SFRP1: secreted frizzled related protein; TEB: terminal end buds; TGF-β, transforming growth factor-β; RANKL: receptor of activated NF-κB ligand; MaSC: mammary stem cell.

## Competing interests

The authors do not have any financial or personal relationships with other people or organizations that could inappropriately influence the work described in this manuscript.

## Authors’ contributions

KG drafted the manuscript and performed all of the described experiments. AS provided our laboratory with the SFRP^−/−^ mice. GC evaluated and assisted in the interpretation of stained tissue slides, and edited the manuscript. SS participated in the study design, edited the manuscript, and gave final approval of the version to be published. All authors read and approved the final manuscript.
